# Role of microRNA‐155 in modifying neuroinflammation and γ‐aminobutyric acid transporters in specific central regions after post‐ischaemic seizures

**DOI:** 10.1111/jcmm.14358

**Published:** 2019-05-29

**Authors:** Wenwen Zhang, Luping Wang, Xiaochuan Pang, Jian Zhang, Yi Guan

**Affiliations:** ^1^ Department of Anesthesiology The First Hospital of Jilin University Changchun Jilin China; ^2^ Department of Anesthesiology School and Hospital of Stomatology, Jilin University Changchun Jilin China; ^3^ Clinical Laboratory The First Hospital of Jilin University Changchun Jilin China; ^4^ Department of Neurosurgery The First Hospital of Jilin University Changchun Jilin China

**Keywords:** cerebral ischaemia, GABA, miRNA‐155, neuroinflammation, seizure

## Abstract

In the central nervous system, interleukin (IL)‐1β, IL‐6 and tumour necrosis factor (TNF)‐α have a regulatory role in pathophysiological processes of epilepsy. In addition, γ‐aminobutyric acid (GABA) transporter type 1 and type 3 (GAT‐1 and GAT‐3) modulate the levels of extracellular GABA in involvement in the neuroinflammation on epileptogenesis. Thus, in the current report we examined the effects of inhibiting microRNA‐155 (miR‐155) on the levels of IL‐1β, IL‐6 and TNF‐α, and expression of GAT‐1 and GAT‐3 in the parietal cortex, hippocampus and amygdala of rats with nonconvulsive seizure (NCS) following cerebral ischaemia. Real time RT‐PCR, ELISA and Western blot analysis were used to examine the miR‐155, proinflammatory cytokines (PICs) and GAT‐1/GAT‐3 respectively. With induction of NCS, the levels of miR‐155 were amplified in the parietal cortex, hippocampus and amygdala and this was accompanied with increases of IL‐1β, IL‐6 and TNF‐α. In those central areas, expression of GAT‐1 and GAT‐3 was upregulated; and GABA was reduced in rats following NCS. Intracerebroventricular infusion of miR‐155 inhibitor attenuated the elevation of PICs, amplification of GAT‐1 and GAT‐3 and impairment of GABA. Furthermore, inhibition of miR‐155 decreased the number of NCS events following cerebral ischaemia. Inhibition of miR‐155 further improved post‐ischaemia‐evoked NCS by altering neuroinflammation‐GABA signal pathways in the parietal cortex, hippocampus and amygdala. Results suggest the role of miR‐155 in regulating post‐ischaemic seizures via PICs‐GABA mechanisms.

## 1 INTRODUCTION

Cerebral ischaemia is considered as one of the great risk factors for progress of seizures and the incidence of seizures induced by post‐ischaemia presently tends to increase.^1^ It has been reported that post‐ischaemic stroke seizures lead to excitotoxicity, which evokes mitochondrial dysfunction, neuroinflammation and oxidative stress resulting in neuronal cell death.^2^, ^3^ Notably, these conditions have substantial pathophysiological impacts on ischaemic stroke progression and this thereby deteriorates prognosis in patients.^4^ Nevertheless, the underlying mechanisms leading to provoked post‐ischaemic seizures remain to be determined.

Two main subtypes of γ‐aminobutyric acid (GABA) transports (GATs), namely GAT‐1 and GAT‐3, are responsible for the control of central extracellular GABA levels.^5^, ^6^ In the central nervous system (CNS), these transporters appear in neuronal cells (predominantly GAT‐1) and glial cells (predominantly GAT‐3), and the prior reports have revealed the role of GATs in modifying GABA receptor‐mediated postsynaptic tonic and phasic inhibition in the cerebral cortex, hippocampus, etc.^5^, ^6^ It is well known that imbalanced inhibitory (GABA) and excitatory (glutamate) synaptic neurotransmissions are associated with adjustment of ion channel activity in contribution to regulation of brain functions.^7^, ^8^, ^9^, ^10^ In order to determine the basic role of central GABAergic transmission in the process of epileptic activity following cerebral ischaemia,^11^, ^12^ in the current study, we performed the middle cerebral artery occlusion (MCAO) in rats to induce post‐ischaemic nonconvulsive seizure (NCS) and further determined the protein levels of GAT‐1 and GAT‐3 in the parietal cortex, hippocampus and amygdala.

Proinflammatory cytokines (PICs) (such as interleukin [IL]‐1β, IL‐6 and tumour necrosis factor [TNF]‐α) are increased in plasma, cerebrospinal fluid and neuronal tissues of patients during the progress of on epileptogenesis.^13^, ^14^ This result is in agreement with the effects of PICs on the pathophysiological responses of epilepsy and/or seizure‐induced cerebral damages. Additionally, a prior study has revealed that the protein levels of IL‐1β, IL‐6 and TNF‐α in the parietal cortex, hippocampus and amygdala are considerably elevated during epilepsy evoked by cerebral injection of kainic acid.^15^ Results of this prior study also indicate that GAT‐1 and GAT‐3 are upregulated and epilepsy‐increased IL‐1β, IL‐6 and TNF‐α results in enhanced GAT‐1 and GAT‐3 in those specific brain regions.^15^ Thus, in the current study, we postulated that elevation of IL‐1β, IL‐6 and TNF‐α is accompanied with the greater levels of GAT‐1 and GAT‐3 in the parietal cortex, hippocampus and amygdala of NCS rats after cerebral ischaemia.

MicroRNAs (miRNAs) are small noncoding endogenous RNA molecules that can alter their target mRNA through binding in the message 3′‐UTR.^16^ MicroRNAs have been shown to have important contributions to multiple pathophysiological processes: cellular death and survival, cellular response to stress, stem cell division, pluripotency, etc.^17^ MicroRNAs also play a role in regulating disease processes including cancer, cardiovascular and neurological diseases.^18^, ^19^, ^20^ As a result of their small size, relative ease of delivery and sequence specificity in recognizing their targets, miRNAs have been considered as promising therapeutic targets with respect to drug development.^21^

Among various miRNAs, microRNA‐155 (miR‐155) plays a role in various physiological and pathological processes.^22^, ^23^, ^24^, ^25^ MicroRNA‐155 is involved in chronic immune response by amplifying the proliferative response of T cells via the downregulation of lymphocyte‐associated antigens.^26^ In autoimmune disorders, a higher expression of miR‐155 is observed in patients' tissues and synovial fibroblasts.^24^ In multiple sclerosis, upregulation of miR‐155 has been observed in peripheral nerve and CNS‐resident myeloid cells, blood monocytes in the circulation and stimulated microglia.^27^ Also, a prior study has suggested that miR‐155 is involved in inflammation and upregulation of miR‐155 results in chronic inflammation in human beings.^25^

Nonetheless, it remains unknown for the role of miR‐155 in engagement of post‐ischaemic seizures. Therefore, in the current study, we determined the modulating effects of miR‐155 on IL‐1β, IL‐6 and TNF‐α and GAT‐1 and GAT‐3 expression along with GABA concentrations in the parietal cortex, hippocampus and amygdala of rats after induction of cerebral ischaemia. We hypothesized that inhibition of miR‐155 decreases ischaemia‐activated PICs, and this attenuates upregulation of GABA transporters and thereby stabilizes GABA in the parietal cortex, hippocampus and amygdala. We also hypothesized that inhibition of miRNA‐155 improves NCS following MCAO through PIC‐GABA mechanisms.

## 2 MATERIALS AND METHODS

### 2.1 Animal

All animal experimental procedures were performed in accordance with the guidelines of the International Association for the Study of Pain and they were approved by the Animal Care and Use Committee of Jilin University. Male Sprague‐Dawley rats (200‐250 g) were housed in individual cages with free access to food and water and were kept in a temperature‐controlled room (25°C) on a 12/12 h light/dark cycle.

### 2.2 Lateral ventricle cannulation and administration of miR‐155 inhibitor

The rats were anaesthetized by using sodium pentobarbital (45 mg/kg bodyweight, ip) and after this they were immobilized in a stereotaxic apparatus (David Kopf, USA). Midline incision was made and the skull was exposed and one burr hole was drilled. Following this procedure, cannulation was made in the lateral ventricle with an L‐shaped stainless steel in rats (3.7 mm posterior to the bregma, 4.1 mm lateral to the midline, and 3.5 mm under the dura). The guide cannula was secured to the skull using dental zinc cement. Then, the cannula was connected to an osmotic minipump (Alzet pump brain infusion kit, DURECT Inc, Cupertino, CA) with polycarbonate tubing. The pumps were placed subcutaneously between the scapulae and the pumps were loaded with miR‐155 inhibitor (5′AAU UAC GAU UAG CAC UAU CCC CA‐3′) and its corresponding scramble for negative controls (5 μg in artificial cerebrospinal fluid, Biomics Biotech, Nantong, China) respectively. The inhibitor and scramble were delivered for a period of 24 hours at a rate at 0.25 μL per hour. This intervention allowed us to give continuously drugs via intracerebroventricular (ICV) infusion.

### 2.3 A model of MCAO and experimental groups

One day after ICV infusion of miR‐155 inhibitor and its scramble, NCS was induced by the MCAO. Sodium pentobarbital (45 mg/kg bodyweight, ip) was used to anaesthetize the rats, the right common carotid artery was exposed at the level of external and internal carotid artery bifurcation. The external carotid artery and its branches were closed and cut at the lingual and maxillary artery branches. Through the stump of the external carotid artery, nylon suture (3‐0 monofilament) was inserted into the internal carotid artery. The filament was advanced for ~20 mm into the anterior cerebral artery. During this procedure, a laser‐doppler flowmeter was used to continuously monitor cortical cerebral blood flow. Once a constant decrease in blood flow was seen at ipsilateral side to the occlusion, the filament was secured to the vessel by ligation. Generally, the animals showing a reduction in cerebral blood flow of >70% are considered ischaemia and only those rats were included for data analysis in this report. In sham control rats, the same surgical procedures were conducted without arterial occlusion.

During 24 hours following MCAO, electroencephalogram (EEG) data were collected.^28^ Through burr holes over bilateral frontal and parietal regions of the cortex four electrodes were implanted on skull. A reference electrode was implanted posterior to lambda over the transverse sinus. The electrodes were connected to a multi‐pin connector. EEG data were obtained via a Grass polygraph amplifier and digitizing system (Grass Lab). The NCSs were examined via stainless steel electrodes implanted on the skull of rats. The off‐line EEG traces were analyzed according to NCS criteria.^28^ The number of NCS events over time was considered as the frequency of NCS in each animal.

At the end of recordings the brains were taken out and the levels of PICs, expression of GAT‐1 and GAT‐3 in the parietal cortex, hippocampus and amygdala were assessed. Accordingly, the rats were included in sham control group (n = 12) and MCAO group with scramble (n = 15) and MCAO group with miR‐155 inhibitor (n = 15). In addition groups, 25 rats were used to examine time course of miR‐155 changes after induction of MCAO.

### 2.4 Real‐time PCR

The brain was taken out and the tissues of the parietal cortex, hippocampus and amygdala were obtained under an anatomical microscope. The tissues were processed for the extraction of total RNA (RNeasy Mini Kit; Qiagen, CA). The TaqmanW Universal PCR Master Mix was used to conduct RT‐PCR. For the mRNA amplification, this mix has AmpliTaq GoldW DNA Polymerase, AmpEraseW UNG, ROX passive reference, buffer and dNTPs, and gene‐specific primers. 18s rRNA was also used as an endogenous control to correct for variations in the samples. RT‐PCR was conducted in duplicate in 96‐well plates containing 2 μL of cDNA. The thermal conditions of the cycles were 50°C for 2 minutes, 60°C for 30 minutes and 95°C for 5 minutes and this procedure was followed by 40 cycles at 94°C for 20 seconds and 62°C for 60 seconds. The data were obtained in the ABI PRISM SDS 7000 thermal cycler. The 2^−ΔΔCt^ comparative method was used to obtain relative quantification of target gene expression and the threshold cycle value was determined by the point at which there was a statistically significant amplification in fluorescence.

### 2.5 ELISA measurement

All the tissues from individual rats were sampled for the analysis. In brief, the parietal cortex, hippocampus and amygdala were removed. Total protein was then extracted by homogenizing the hippocampus sample in ice‐cold immunoprecipitation assay buffer with protease inhibitor cocktail kit. The lysates were centrifuged and the supernatants were collected for measurements of protein concentrations using a bicinchoninic acid assay reagent kit.

Interleukin‐1β, IL‐6 and TNF‐α were determined by using a two‐site immunoenzymatic assay (Wuhan Fine Biotech Co). Rabbit anti‐ IL‐1β, anti‐IL‐6 and anti‐TNF‐α antibodies were used to coat polystyrene 96‐well microtiter immunoplates. After being incubated overnight at room temperature and 2 hours of incubation with the coating buffer, plates were washed. The diluted samples and each PIC standard solution were distributed in each plate and left overnight. The plates were washed and incubated with anti‐ IL‐1β, anti‐IL‐6 and anti‐TNF‐α galactosidase. After this, the plates were washed and incubated with substrate solution. After an incubation of 2 hours, the optical density was determined using an ELISA reader with wavelength of 575 nm. Likewise, the levels of GABA were determined by the ELISA methods.

### 2.6 Western blot analysis

A standard Western blot procedure was used to examine expression of GAT‐1 and GAT‐3 in the nerve tissues. Total protein was extracted by homogenizing samples of the parietal cortex, hippocampus and amygdala in ice‐cold buffer. For measurements of protein concentrations, the lysates were centrifuged and the supernatants were then collected. Protein concentrations were assessed using the bicinchoninic acid assay reagent kit. After being denatured in buffer, the supernatant samples containing 20 μg of protein were loaded onto Mini‐PROTEAN TGX gels, transferred to a polyvinylidene fluoride membrane, and incubated with primary antibodies (at 1:500): rabbit anti‐GAT‐1 and rabbit anti‐GAT‐3 (Cat#ab426 and Cat#ab431; Abcam Co.). Next, the membranes were washed and incubated with anti‐rabbit antibody conjugated with an alkaline phosphatase (at 1:1000), and examined by enhanced chemiluminescence. Then the membrane was exposed onto an x‐ray film to examine the recognized bands of the primary antibodies. After this, the film was scanned and the optical density of GAT‐1/GAT‐3 bands was determined by using the NIH Scion Image software.

### 2.7 Statistical analysis

One‐way measures analysis of variance was employed for comparison, followed by Tukey's post hoc test as appropriate. All data were shown as mean ± SEM. For all analyses, statistical significance was set at *P* < 0.05. All statistical analyses were performed by using spss for Windows (version 13, SPSS).

## 3 RESULTS

### 3.1 The levels of miR‐155 after MCAO

**Figure 1:**
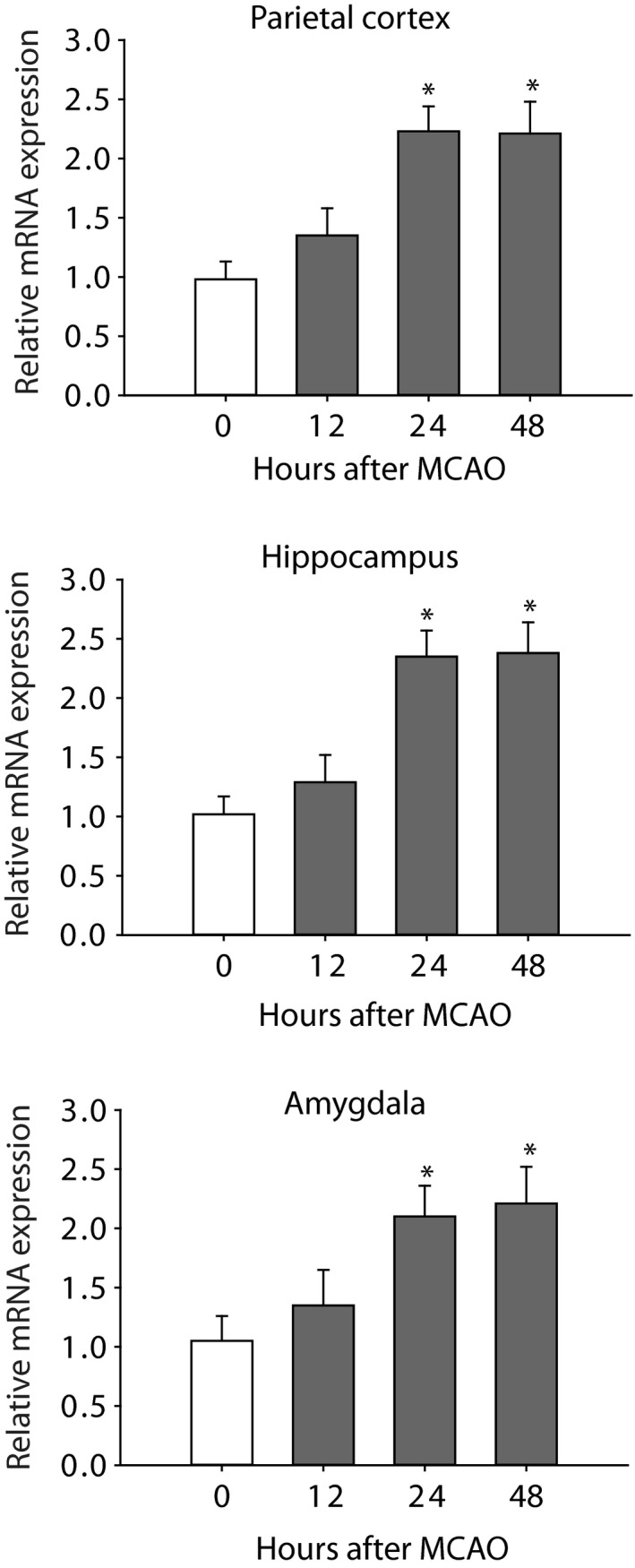
The levels of microRNA‐155 mRNA were increased in the parietal cortex, hippocampus and amygdala 1 d after the end of middle cerebral artery occlusion (MCAO) procedure and the increase was stabilized 24 h after the MCAO procedure and remained at a high level. *P < 0.05 vs its level before the MCAO. Point ‘0’ indicates before MCAO. The number of rats = 8‐12 in each group

In this study, we first examined the time course for changes of miR‐155 after MCAO. Figure 1 shows a stabilized increase in the levels of miR‐155 of the parietal cortex, hippocampus and amygdala 24 hours after the end of MCAO procedure. Based on this result, the time point of 24 hours was selected to examine the effects of miR‐155 inhibitor for the rest of experiments.

### 3.2 The changes of IL‐1β, IL‐6 and TNF‐α

**Figure 2:**
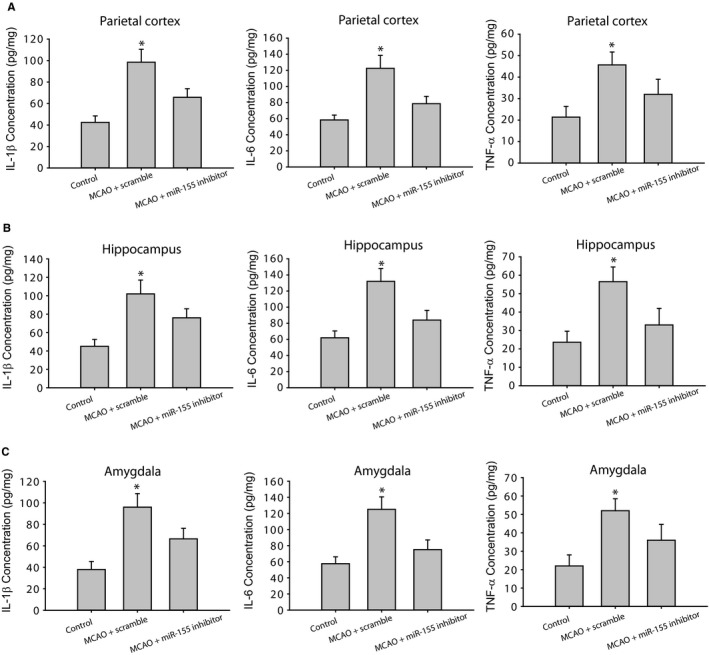
The levels of IL‐1β, IL‐6 and TNF‐α in the parietal cortex (A), hippocampus (B) and amygdala (C). The cytokines were significantly increased in middle cerebral artery occlusion (MCAO) rats with scramble as compared with control animals. MicroRNA‐155 (MiR‐155) inhibitor attenuated increases of those cytokines in those brain regions of MCAO rats. *P < 0.05, indicated rats with MCAO with scramble (n = 15) vs control rats (n = 12) and MCAO rats with miR‐155 inhibitor (n = 15)

Figure 2 (A‐C) demonstrates that IL‐1β, IL‐6 and TNF‐α were considerably amplified in the parietal cortex, hippocampus and amygdala of rats with MCAO (*P* < 0.05, MCAO rats with scramble/n = 15 vs control rats/n = 12) in comparison with control group. As MCAO rats were given with miR‐155 inhibitor (n = 15), elevation of IL‐1β, IL‐6 and TNF‐α was attenuated (*P* < 0.05, MCAO rats with miR‐155 inhibitor vs MCAO rats with scramble, n = 15 in each group).

### 3.3 GAT‐1 and GAT‐3

**Figure 3:**
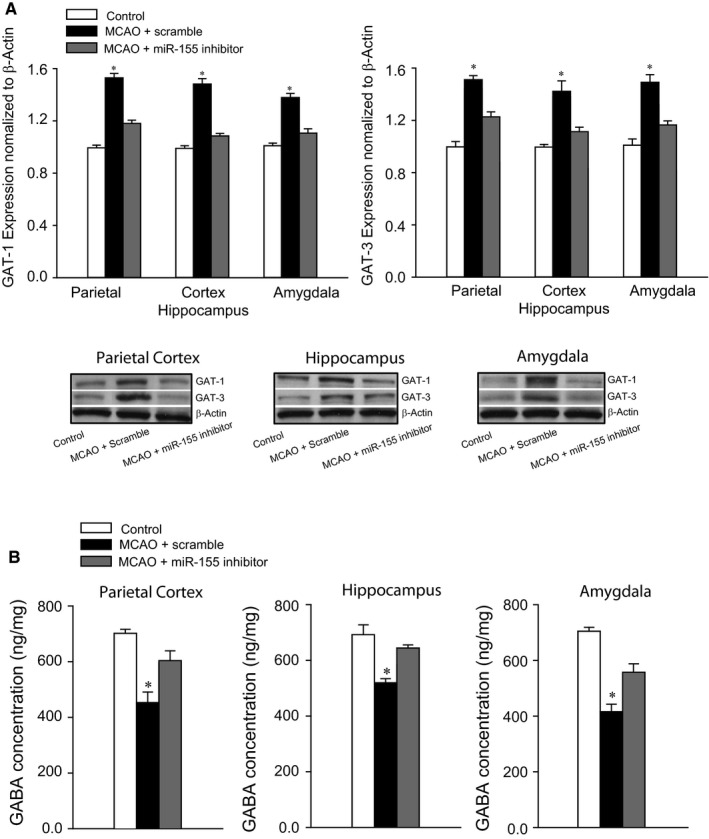
A, Expression of γ‐aminobutyric acid (GABA) transporter type 1 (GAT‐1) and type 3 (GAT‐3) in the parietal cortex, hippocampus and amygdala. GAT‐1 and GAT‐3 were significantly increased in middle cerebral artery occlusion (MCAO) rats with scramble (n = 15) as compared with control animals (n = 12). Intracerebroventricular (ICV) injection of microRNA‐155 (MiR‐155) inhibitor attenuated enhanced GAT‐1 and GAT‐3 induced by MCAO (n = 15). *P < 0.05 vs control rats and rats with miR‐155 inhibitor. Top panel: averaged data; and bottom panel: typical bands are representative of expression of GAT‐1 and GAT‐3 in the different regions of three groups of rats. B, The levels of GABA in the parietal cortex, hippocampus and amygdala. The levels of GABA were diminished in MCAO rats as compared with control animals. MicroRNA‐155 inhibitor restored impairment of GABA in these brain regions. *P < 0.05, indicated rats with MCAO with scramble (n = 15) vs control rats (n = 12) and MCAO rats with ICV injection of miR‐155 inhibitor (n = 15)

Figure 3A illustrates greater expression of GAT‐1 and GAT‐3 in the parietal cortex, hippocampus and amygdala of MCAO rats (n = 8‐12, *P* < 0.05 vs control animals) in comparison with control rats (n = 6‐10). Likewise, application of miR‐155 inhibitor attenuated GAT‐1 and GAT‐3 expression (*P* < 0.05 vs MCAO animals with scramble). Nevertheless, there were insignificant differences in GAT‐1 and GAT‐3 in control rats and MCAO rats with ICV injection of miR‐155 inhibitor (*P* > 0.05 between two groups).

### 3.4 Levels of GABA

Figure 3B reveals that the levels of GABA were less in the parietal cortex, hippocampus and amygdala of MCAO rats (*P* < 0.05 vs control rats; n = 15 in MCAO group; and n = 12 in control group) in comparison with control group. As miR‐155 inhibitor was infused in MCAO rats (n = 15), downregulation of GABA levels was largely restored (*P* < 0.05 vs rats with ICV miR‐155 scramble).

### 3.5 Incidence of NCS

**Figure 4:**
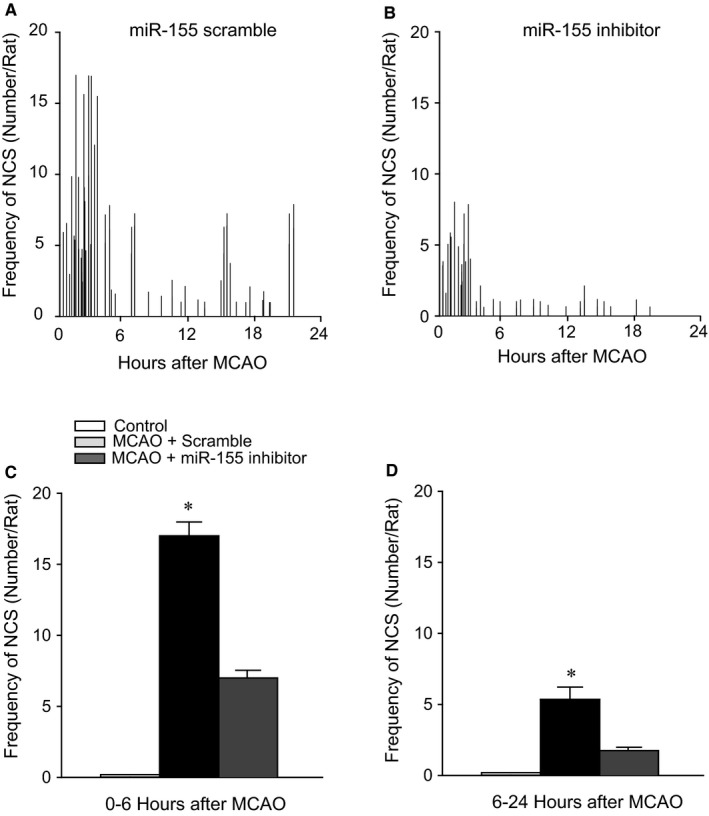
The frequency of nonconvulsive seizure (NCS) events of representative animals receiving microRNA‐155 (miR‐155) scramble (A) and miR‐155 inhibitor (B) following induction of middle cerebral artery occlusion (MCAO). The number of NCS events on hour basis is presented. Initial NCS events appeared within 0‐6 h following induction of MCAO and continued within 24 h. MiR‐155 inhibitor significantly reduced NCS frequency. (C,D) Averaged data showing the effects of miR‐155 inhibitor on NCS events within 6 and 6‐24 h after the end of MCAO. *P < 0.05, indicated MCAO rats with treatment of miR‐155 scramble (n = 15) vs MCAO rats with miR‐155 inhibitor (n = 15). Note that there were no NCS events observed in control rats (n = 12)

Nonconvulsive seizure was not observed in control rats (n = 12). Rats showed to have NCS events following MCAO with administration of miR‐155 scramble. Figure 4 demonstrates that ICV injection of miR‐155 inhibitor reduced the number of NCS incidence in cerebral rats. Middle cerebral artery occlusion resulted in a less NCS incidence in rats with miR‐155 inhibitor in comparison with rats infused with scramble (*P* < 0.05, miR‐155 inhibitor vs scramble in MCAO rats; n = 15 in each group).

## 4 DISCUSSION

In the current study, we showed that inhibition of miR‐155 decreases ischaemia‐activated PICs, and this attenuates upregulation of GABA transporters and thereby stabilizes GABA in the parietal cortex, hippocampus and amygdala. We further showed that inhibition of miRNA‐155 improves NCS following MCAO through PIC‐GABA mechanisms.

Neuroinflammation is involved in changes of neuronal damage and electronic encephalography of epileptic activity and following induction of seizure amplification of IL‐1β, IL‐6 and TNF‐α in hippocampus are found.^29^ A prior report further showed that IL‐1β, IL‐6 and TNF‐α are increased in the parietal cortex, hippocampus and amygdala during seizure in rats.^15^ Data of this prior study also suggest that GABA transporters (GAT‐1 and GAT‐3) are increased during seizure and the effects are likely through receptor mechanisms of IL‐1β and TNF‐α.^15^ On the basis of evidence, in this report we determined the effects of MCAO on IL‐1β, IL‐6 and TNF‐α along with expression of GAT‐1 and GAT‐3 in the brain during evoked NCS. We further determined if ICV infusion of miR‐155 inhibitor altered PICs, GAT‐1/GAT‐3 and GABA. Our data offered the solid evidence that cerebral administration of miR‐155 inhibitor alleviates deficiency of GABA in the parietal cortex, hippocampus and amygdala likely through decreasing IL‐1β, IL‐6 and TNF‐α along with GAT‐1 and GAT‐3.

Increases of PICs in the CNS are related to seizure vulnerability and seizure‐induced pathological changes,^30^, ^31^, ^32^ indicating that neuroinflammation is engaged in the process of epileptogenesis. In epilepsy, neural injuries in the CNS are observed in rats and this is augmented by upregulation of PICs (IL‐1β, IL‐6 and TNF‐α) in glial cells.^29^, ^33^ Additionally, neuronal degeneration is detected as a result of increases of PICs.^34^, ^35^ For example, IL‐1β increases seizure susceptibility in rat brains.^36^ Intracerebral injection of IL‐1β leads to limbic seizures in wild‐type mice, but not in transgenic mice with deficient IL‐1β receptors.^37^ Furthermore, after thalidomide is used to decrease the levels of TNF‐α, antiepileptic activity is seen after thalidomide.^38^

In the CNS, GABA is the major inhibitory neurotransmitter in regulation of neuronal excitability. After GABA is released from presynaptic terminals, GATs can remove extracellular GABA,^7^, ^9^ which thereby results in termination of inhibitory synaptic transmission. This signal pathway also leads to GABA spillover to neighbouring synapses 7, 8 and thereby excessive tonic activation of synaptic and extrasynaptic GABA receptors are stopped and then GABA homeostasis is maintained.^7^, ^10^ In contrast, the GATs functions are impaired under certain pathophysiological conditions, which leads to the exaggerated GABA release.^39^, ^40^ Data of this report demonstrated that IL‐1β, IL‐6 and TNF‐α were significantly augmented in the parietal cortex, hippocampus and amygdala after cerebral ischaemia was induced. Our results further demonstrated that GAT‐1 and GAT‐3 were amplified in the central areas of cerebral ischaemic rats in comparison with controls. Consistently, our data demonstrated that GABA was reduced in those central areas of ischaemic rats. Main findings from the current report are that ICV infusion of miR‐155 inhibitor attenuated amplification of IL‐1β, IL‐6 and TNF‐α along with increases of GAT‐1 and GAT‐3 and thus restored impairment of GABA in those brain regions. Moreover, inhibition of miR‐155 reduced the incidence of NCS events amplified by MCAO.

Accumulated evidence has demonstrated critical roles played by various miRNAs in modifying pathophysiological processes with animal models and clinical ischaemic disorders.^41^, ^42^, ^43^ Specifically, it was reported that anti‐inflammatory protein suppressor of cytokine signalling 1 (SOCS1) is one of the target genes of miR‐155.^44^ Suppressor of cytokine signalling 1 is a critical regulator of inflammation and negatively regulates the feedback of inflammation.^45^ Deficiency of SOCS1 results in amplified responsiveness to inflammation in cells and/or in animals.^46^, ^47^ MicroRNA‐155 is also involved in regulating stroke development by promoting expression of TNF‐α and IL‐1β and by decreasing SOCS1.^48^, ^49^ Thus, more investigations are needed to explain the networks of miR‐155 in involvement of seizure development because of cerebral ischaemia.

Our data suggest that cerebral ischaemia increases IL‐1β, IL‐6 and TNF‐α in the parietal cortex, hippocampus and amygdala, and this causes upregulation of GAT‐1 and GAT‐3 and decreases of GABA through stimulation of PIC signal pathways. Consequently, GABA‐arbitrated inhibitory effects on neuronal activities in those central areas are likely impaired and this is further to lead to neurological deficits followed after cerebral ischaemia. Notably, cerebral inhibition of miR‐155 improves abnormal PIC‐GABA signal pathways. Because GAT‐1 is extensively expressed in neuronal cells and GAT‐3 is mainly expressed in glial cells of the CNS,^5^, ^7^ data of the present report indicate that miR‐155 plays a regulatory role in GABA inhibitory systems in the neuronal and glial cells after MCAO‐evoked NCS. Blocking miR‐155 has beneficial effects on MCAO‐induced NCS events via PIC‐GABA signal.

In conclusion, our evidence shows that cerebral ischaemia increases incidence of NCS, and during this process, IL‐1β, IL‐6 and TNF‐α are amplified in the parietal cortex, hippocampus and amygdala. Cerebral ischaemia further regulates GAT‐1 and GAT‐3 and thus reduces GABA in those central areas. These abnormalities augment neuronal excitability in the CNS of animals. Notably, ICV administration of miR‐155 reduces PICs, attenuates expression of GAT‐1 and GAT‐3 and alleviates impairment of GABA. This also alleviates NCS incidence induced by cerebral ischaemia. Data may provide evidence for treatment of symptoms in patients with epilepsy after cerebral ischaemia.

## CONFLICT OF INTEREST

None.
